# *Bacillus subtilis* Synthesized Iron Oxide Nanoparticles (Fe_3_O_4_ NPs) Induced Metabolic and Anti-Oxidative Response in Rice (*Oryza sativa* L.) under Arsenic Stress

**DOI:** 10.3390/toxics10100618

**Published:** 2022-10-18

**Authors:** Sehresh Khan, Nazneen Akhtar, Shafiq Ur Rehman, Shaukat Shujah, Eui Shik Rha, Muhammad Jamil

**Affiliations:** 1 Department of Biotechnology and Genetic Engineering, Kohat University of Science & Technology (KUST), Kohat 26000, Pakistan; 2 Department of Biology, University of Haripur, Haripur 22620, Pakistan; 3 Departments of Chemistry, Kohat University of Science & Technology (KUST), Kohat 26000, Pakistan; 4 Department of Well-Being Resources, Sunchon National University, Suncheon 540-742, Korea

**Keywords:** arsenic, iron oxide nanoparticles, toxicity, oxidative stress, *Oryza Sativa* L.

## Abstract

Nanoparticle (NP) application is most effective in decreasing metalloid toxicity. The current study aimed to evaluate the effect of *Bacillus subtiles* synthesized iron oxide nanoparticles (Fe_3_O_4_ NPs) against arsenic (As) stress on rice (*Oryza sativa* L.) seedlings. Different concentrations of As (5, 10 and 15 ppm) and *Bacillus subtilis* synthesized Fe_3_O_4_ NPs solution (5, 10 and 15 ppm) alone and in combination were applied to rice seedlings. The results showed that As at 15 ppm significantly decreased the growth of rice, which was increased by the low level of As. Results indicated that *B. subtilis* synthesized Fe_3_O_4_ NP-treated plants showed maximum chlorophyll land protein content as compared with arsenic treatment alone. The antioxidant enzymes such as SOD, POD, CAT, MDA and APX and stress modulators (Glycine betain and proline) also showed decreased content in plants as compared with As stress. Subsequently, *Bacillus subtilis* synthesized Fe_3_O_4_ NPs reduced the stress associated parameters due to limited passage of arsenic inside the plant. Furthermore, reduction in H_2_O_2_ and MDA content confirmed that the addition of *Bacillus subtilis* synthesized Fe_3_O_4_ NPs under As stress protected rice seedlings against arsenic toxicity, hence enhanced growth was notice and it had beneficial effects on the plant. Results highlighted that Fe_3_O_4_ NPs protect rice seedlings against arsenic stress by reducing As accumulation, act as a nano adsorbent and restricting arsenic uptake in rice plants. Hence, our study confirms the significance of *Bacillus subtilis* synthesized Fe_3_O_4_ NPs in alleviating As toxicity in rice plants.

## 1. Introduction

Arsenic (As) pollution in ground water and soil is a worldwide problem, which is increased by rapid industrialization [1]. Among metals, As is considered as one of the most toxic metalloids and their common sources includes herbicides, insecticides, fertilizers, wood preservatives, mining, smelting and coil combustion [2]. Arsenic released from these sources causes different hazardous impacts on humans, animals and plants [3]. From these sources, natural and anthropogenic sources are them a in causes of As contamination. Arsenic-contaminated crops show a high level of arsenic threat to human health due to a high accumulation of As. Inorganic forms of arsenic are more dominant in water and soil. There is a lack of proper disposal of arsenic that results in health risks to populations with a high rice consumer rate [4,5,6]. Arsenic is a redox- sensitive trace element and exists in different oxidation states such as As III, V and 0. Arsenic accumulation in plants causes physiological changes that hamper plant growth and productivity [7] Arsenic (III) reacts with tissue proteins and the sulfhydral group of enzymes, leading to inhibition of cellular function and, at last, cell death. Arsenic (V) is phosphate analogous and enters through the phosphate transporter, interferes with different cellular processes in plants, such as ATP synthesis and oxidative phosphorylation [8].

Arsenic disturbs plant physiological, morphological, biochemical and molecular processes [9]. To cope with arsenics tress and different organic solutes, as sophisticated and complex set of mechanisms are involved in rice plants under arsenic stress. The well-known metabolic response in rice plants under arsenic stress is proline accumulation, which is generally an indicator of dehydration and susceptibility of plants with arsenic stress. The function of proline is to stabilize the structure, maintain the osmotic adjustment and free radical scavengers in rice plants. Soluble sugar maintains the water content and osmotic adjustment in rice plants, but under arsenic stress, soluble sugar content decreases or increases in plants [10,11,12,13,14].

In recent years, among different technologies, nanotechnology has emerged as an important tool in improving agricultural productivity. It was demonstrated that in spinach plants’ growth rates were boosted by TiO_2_ NPs [15,16]. Iron oxide NPs increased the physiological activity in *Arachis hypogaea,* wheat and *Glycine max* seed lings [17,18,19]. Chlorophyll production is boosted by silver nanoparticles without showing any toxic impact on the *Brassica* plant, and [20,21] also investigated nanoparticles’ concentration-dependent impact on barley plants. It has been reported that NPs have a strong toxic effect on plants, which depends on the dose and nature of nanoparticles [22,23,24,25,26,27]. Silver NPs have a positive role in the promotion of plant growth and enable the plant to defend against pathogens [28,29]. It was reported in spinach that exposure of TiO_2_ NPs has significant effects on chlorophyll content, nitrogen metabolism and plant growth [30]. It was demonstrated that photosynthetic content increases with AgNPs in the *Brassica* plant [31].

The beneficial role of iron against arsenic is well documented in plants. It plays a vital role in metabolism [32] and physiological processes of the plants. Iron is necessary for chlorophyll l synthesis, DNA replication, scavenging ROS and the electron transport chain in chloroplast and mitochondria. In our previous studies, we had synthesized iron oxide nanoparticles (Fe_3_O_4_ NPs) from *Bacillus subtitles* and observed its effect on seed germination under arsenic stress. The current study was designed to investigate the effect of *Bacillus subtilis*-synthesized iron oxide (Fe_3_O_4_ NPs) on the metabolic and anti-oxidative parameters of rice (*Oryza sativa* L.) seedlings under arsenic stress.

## 2. Materials and Methods

### 2.1. Experimental Method and Plant Growth

Rice seeds (*Oryza sativa* L.) were collected from the NARC (National Agriculture Research Center), Islamabad, Pakistan. Arsenic was obtained from Sigma-Aldrich exists as arsenious acid (As III) with a valence state of (+3). Seeds were sterilized with a 3% solution of sodium hypochlorite for 3 min and washed thoroughly with distilled water. After sterilization, healthy seeds were placed in Petri plates under distal water in dark conditions. Seeds were allowed to germinate for 5 days. Seedlings were transferred to plastic trays containing Hoagland media for 3 weeks. After the 3rd week, different concentrations of As (5, 10 and 15 ppm) and *Bacillus* subtilis-synthesized Fe_3_O_4_ NPs solution (5, 10 and 15 ppm) were applied for a period of 1 week. Plants from the control and the treated seedlings were harvested after 28 days and different parameters were analyzed.

### 2.2. Cellular Injury in Rice Plant

Cellular injury in plants was determined by following the protocol of Hamim et al. [33]. Leaves were cut into 10 fine strips of 1 cm and placed in a glass tube containing distilled water. Tubes were incubated at 10 °C for 14 h and the electrical conductivity (C1) was measured, then tubes were autoclaved at 121 °C for 20 min and the electrical conductivity (C1) was measured using a (BMS) conductivity meter, EC-4001. C1/C1 × (100).

### 2.3. Photosynthetic Pigment

The chlorophyll content in the plant was determined by the method of Li et al. [34]. Dry leaf samples (15 mg) and an equal amount of MgO (15 mg) were added in to a tube. Pigment extract ion was carried out by adding 5 mL of methanol and was mixed at 100 rpm in a shaker for 1 h. Samples were centrifuged at 4000 rpm for 5 min. Absorbance was recorded at 470,653, and 666 nm wavelengths using a spectrophotometer. Methanol was used as a blank.

### 2.4. Metabolic Studies

#### 2.4.1. Total Soluble Sugar (TSS)

Total soluble sugar was determined by using the phenol-sulphuric acid method [35]. The fresh plant (50 mg) was mixed in a 90% (3 mL) pre-warmed mixture of ethanol and incubated at 80 °C for 1 h. In the mixture, phenol (5%) and concentrated sulphuric acid were added, and then incubated for 30 min. The absorbance was measured at 485 nm by using filtered distilled water as blank. Soluble sugar content was determined by using a standard curve.

#### 2.4.2. Total Soluble Protein (TSP)

Total soluble protein (TSP) was determined through the Bradford assay (1976) by using BSA as a standard [36]. Fresh samples (500 mg) were mixed with phosphate buffer (10 mL), and the pH was adjusted to 7.8. Tubes were centrifuged at 14,000 rpm for 10 min. In to the reaction solution, 10 µL of protein extract was added and placed at room temperature for five minutes. The absorbance of the mixture was measured at 595 nm by using distilled water as a blank.

#### 2.4.3. Stress Related Parameter (Proline)

Proline content in plants was determined by the methodology of Bates et al. [37]. The salfo salicylic acid 3% (5 mL) was used for crushing the plant samples (100 mg) and centrifugation at 4000 rpm for 30 min. The supernatant was reacted with ninhydrin. The mixture was incubated which has 30 mL of glacial acetic acid along with 10 mL of 6 M phosphoric acid and placed at 100 °C for 1 h. Toluene was used for extraction, and the absorbance was measured at 510 nm. Standard curve was used to estimate the proline content.

#### 2.4.4. Glycine Betain

The methodology of Beauchamp and Fridovich [38] was used to analyze the glycine betain content. Dry plant material (50 mg), 4 mL of de ionized water and equal volumes of Na H_2_SO_4_ (1 mL) were mixed well and kept chilled on ice bags for one hour. Potassiumtri-iodide (Kl_3_) of 0.1 mL was added and vortexes for 3–5 min. After centrifugation, crystals were dissolved in 1, 1-Dichloroethane. The absorbance was recorded at 365 nm by estimating the standard curve.

### 2.5. Antioxidant (SOD, POD, CAT and APX) Determination

Fresh plant material (500 mg) was crushed in 10 mL pre-chilled phosphate buffer (NaH_2_PO_4_. 1H_1_ 00.6663 g/L, Na_2_HPO_4_. 1H_2_O 16.385 g/L). The homogenate sample was centrifuged at 10,000 rpm (4 °C) for 10 min as suggested by Velikova et al. [39].

#### 2.5.1. Superoxide Dismutase (SOD)

Super oxide dismutase was measured in terms of its capacity to slow down the photochemical reduction of NBT as used by previously [40]. For preparation of the substrate, the mixture contained nitro blue tetrazolium (NBT), riboflavin, sodium ethylene diamine tetra acetic acid (Na EDTA) and methionine were used. Reaction tubes containing reaction substrate (3 mL), H_2_O_2_ and enzyme extract were kept under 4000 lux for 10 min. The absorbance was determined at 560 nm [40].

#### 2.5.2. Peroxidase (POD)

Peroxidase activity in control and treated plants was carried out by using the methodology of Li et al. [41]. The assay was performed with crude enzyme extract, Guaiacol, hydrogen peroxide (H_2_O_2_) and Potassium Phosphate Buffer (PBS). The absorbance was recorded at 470 nm [42] and activity was estimated by the method of [43].

#### 2.5.3. Catalase (CAT)

The reaction mixture consisted of enzyme extract; potassium phosphate buffer (PBS) and hydrogen peroxide (H_2_O_2_).The absorbance was measured at 140 nm [44].

#### 2.5.4. Ascorbate Peroxidase (APX)

Ascorbate peroxidase activity was determined by using the methodology of Sofo et al. [45]. The assay was performed with Potassium Phosphate Buffer (PBS), Sodium Ethylene Di amine Tetra Acetic Acid (EDTA), ascorbic acid, hydrogen peroxide and enzyme extract. The absorbance was measured at 190 nm [46].

### 2.6. Stress Bio-Markers

#### 2.6.1. Malondialdehyde (MDA)

The malondialdehyde (MDA) content was determined by taking the reaction mixture of thio-barbituric acid (TBA) and tri-chloroacetic acid (TCA) by following the method of Velikova et al. [39]. After centrifugation at 1519 rpm, MDA content was determined by taking the absorbance at 600 nm and 531 (coefficient of 155 mM^−1^ cm^−1^) [47].

#### 2.6.2. Hydrogen Peroxide

Hydrogen peroxide activity was carried out according to the methodology of Nankano and Asada [48]. The reaction mixture consisted of potassium iodide (KI), potassium phosphate buffer (PBS) and enzyme extract. The absorbance of the mixture was determined at 390 nm.

### 2.7. Determination of Arsenic Content in Plant Parts (Leaf, Shoot and Root)

An atomic absorption spectrophotometer was used to determine the amount of arsenic in different parts of the plant (leaf, shoot and root) [49]. Sulfuric acid and concentrated HNO_3_ were used to digest dried plant parts. The solution was then diluted with 20 mL of distilled water after being heated on a hotplate until it became transparent. The content of arsenic was determined using the protocol [49].

### 2.8. Statistical Analysis

Analysis of variance (ANOVA) was used to determine whether the means were different, considering *p* < 0.05 as a significant level. Duncan’s multiple range tests were performed to determine the least significant difference (LSD) between treatments. All pair-wise comparisons and standard deviation (S.D) of replicates were determined.

## 3. Results

### 3.1. Cell Injury

**Figure 1:**
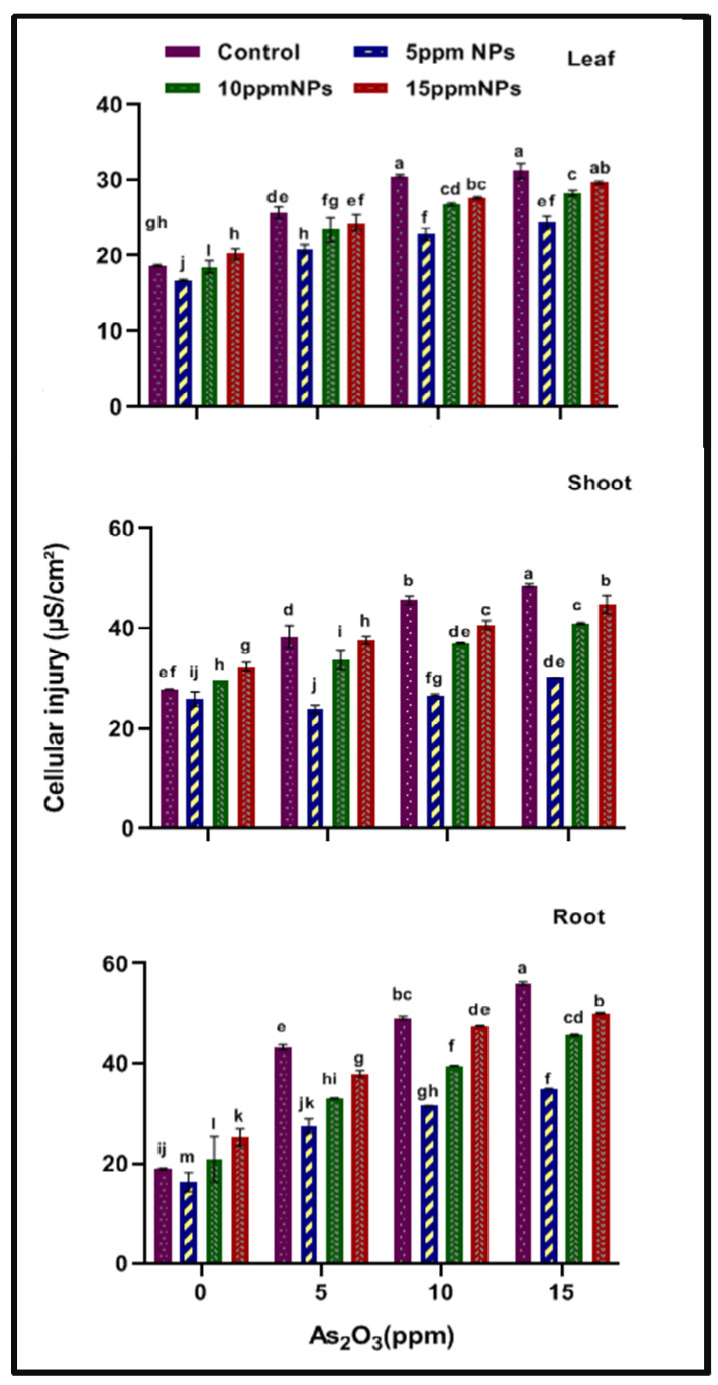
Effect of *Bacillus subtilis*-synthesized iron oxide nanoparticles on the cellular injury of rice (*Oryza sativa* L.) in arsenic contaminated water. Different letters indicate significant differences (*p* < 0.05) between treatments.

The cell injury in the rice plants was increased as arsenic concentrations increased from 5 to 15 ppm. In super basmati at 15 ppm of arsenic stress, maximum cell injury (56.07, 48.54 and 31.20 µS/cm^2^) was noted in the root, shoot and leaf as compared to control (18.96, 27.81 and 18.74 µS/cm^2^). *Bacillus subtilis*-synthesized Fe_3_O_4_ Ns solution minimized the injury level by reducing the value at 15 As + 5 NPs solution (34.98, 30.09 and 24.40 µS/cm^2^) as compared to arsenic stress (Figure 1).

### 3.2. Total Chlorophyll Content

The results revealed that the photosynthetic pigments (chlorophyll a, chlorophyll b and carotenoid) decreased under arsenic stress. The chlorophyll content (a, b and carotenoid) in leaves (2.05, 1.15 and 0.66 mg/g) and shoots (1.74, 1.11 and 0.55 mg/g) was reduced in As-treated plants at 15 ppm as compared to control leaves (3.78, 2.44 and 1.54 mg/g) and shoots (3.35, 1.55 and 1.34 mg/g). The addition of Fe_3_O_4_ NPs (5 ppm) significantly decreased the As toxicity and increased the chlorophyll content in leaves (4.45, 2.85 and 1.83 mg/g) and shoots (3.85, 2.42 and 1.47 mg/g) as compared to the As-alone treatment (Table 1).

### 3.3. Metabolic Studies

#### 3.3.1. Total Soluble Sugar (TSS)

**Figure 2:**
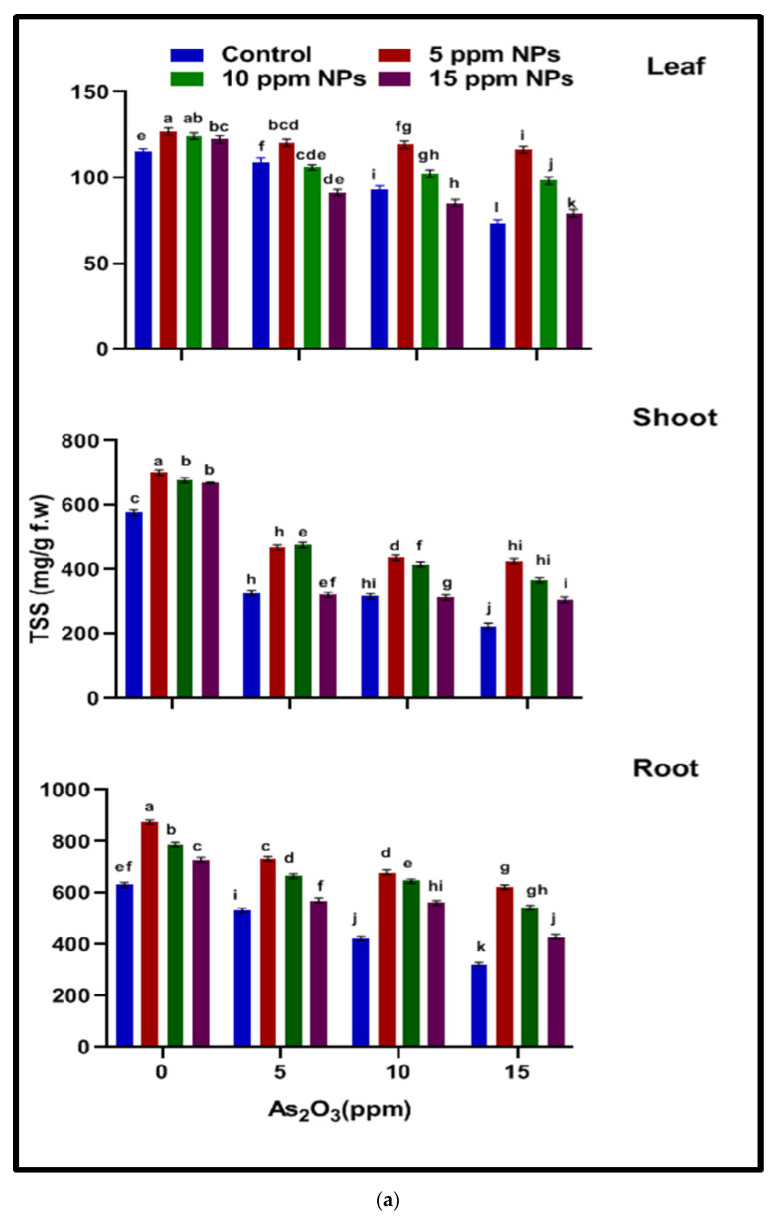

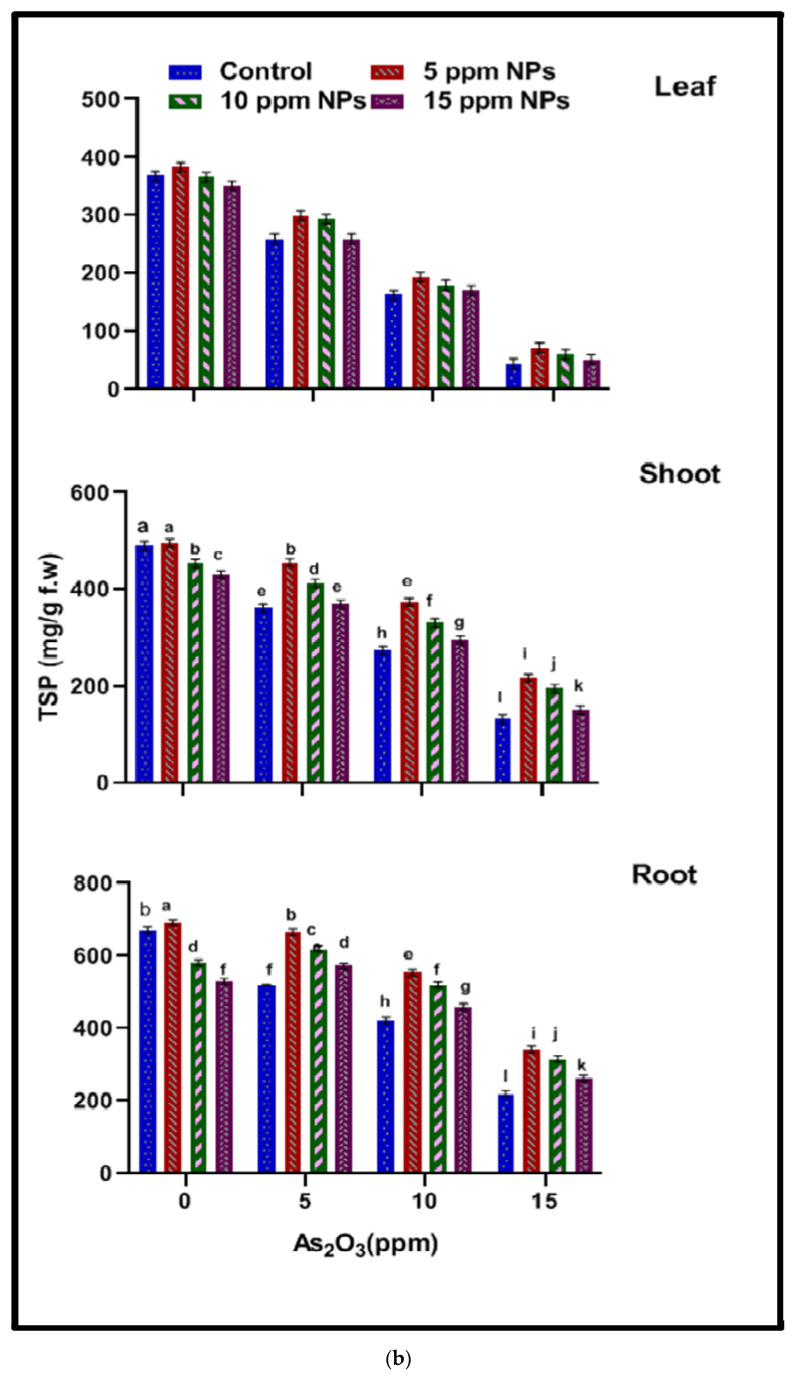
Effect of *Bacillus subtilis*-synthesized iron oxide nanoparticles on TSS (**a**) and TSP (**b**) content of rice (*Oryza sativa* L.) in arsenic contaminated water. Different letters indicate significant differences (*p* < 0.05) between treatments.

The content of total soluble sugar showed an increasing trend in rice plants under an increase in arsenic concentration (5, 10 and 15 ppm). It was determined that the total soluble sugar content in the root, shoot and leaf increased (323.88, 223.88 and 73.38 mg/g f.wt) at 15 ppm under arsenic-induced stress as compared to control (630.87, 576.87 and 115 mg/g f.wt), respectively. Whereas *Bacillus subtilis* synthesized the Fe_3_O_4_ NPs solution at 5 ppm with As 15 ppm decreased the level of TSS in the root, shoot and leaf (621.89, 425.87 and 116.37 mg/g f.wt) as compared to the arsenic-alone treatment (Figure 2).

#### 3.3.2. Total Soluble Protein (TSP)

It was determined that the content of total soluble protein increased in plants under arsenic stress. Total soluble protein content was increased in the root, shoot and leaf (219.67, 132.17 and 44.67 µg/gm) under arsenic-induced stress at 15 ppm, respectively, as compared to control (671.16, 490.66 and 367.67 µg/gm), where as *Bacillus subtilis* synthesized the Fe_3_O_4_ NPs solution with As (5 Fe_3_O_4_ NPs + 15 As) decreased the level of TSP in the root, shoot and leaf (342.66, 217.66 and 71.66 µg/gm) as compared to the arsenic treatment (Figure 2).

#### 3.3.3. Stress-Related Parameter (Proline)

**Figure 3:**
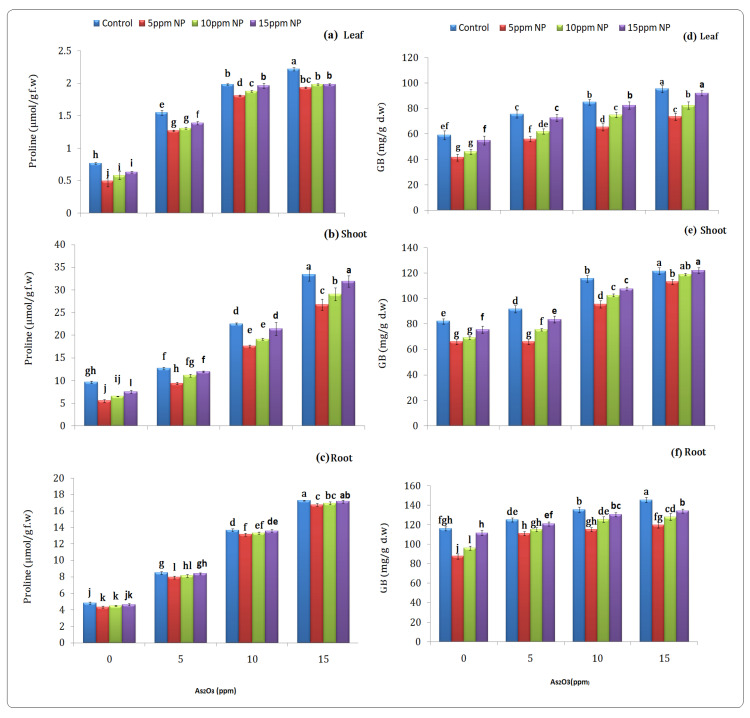
Effect of *Bacillus subtilis* synthesized iron oxide nanoparticles on proline (**a**–**c**) and Glycine betain (**d**–**f**) content of rice (*Oryza sativa* L.) in arsenic-contaminated water. Different letters indicate significant differences (*p* < 0.05) between treatments.

Proline serves as an osmo-protectant and its content increased in rice with the increase in the arsenic concentration. Proline content increased in the root, shoot, and leaf at 15 ppm arsenic stress (17.26, 33.39 and 2.21 µmol/g) as compared to the control (4.79, 9.67 and 0.77 µmol/g) and decreased under 5 ppm *Bacillus subtilis*-synthesized Fe_3_O_4_ NPs solution in the root, shoot, and leaf (4.33, 5.54 and 0.49 µmol/g) as compared to the As-alone treatment (Figure 3).

#### 3.3.4. Glycine Betain

It was observed that glycine betain content in the root, shoot and leaf increased at 15 ppm under arsenic stress (145.58, 121.58 and 95.58 mg/g d.wt) as compared to the control (116.08, 82.08 and 59.08 mg/g d.wt), respectively. Whereas *Bacillus subtilis*-synthesized Fe_3_O_4_ NPs solution with As at 5 ppm decreased the level of GB in the root, shoot and leaf (87.59, 66.09 and 41.58 mg/g d.wt) as compared to the arsenic-alone treatment (Figure 3).

### 3.4. Antioxidant (SOD, POD, CAT and APX) Determination

**Figure 4:**
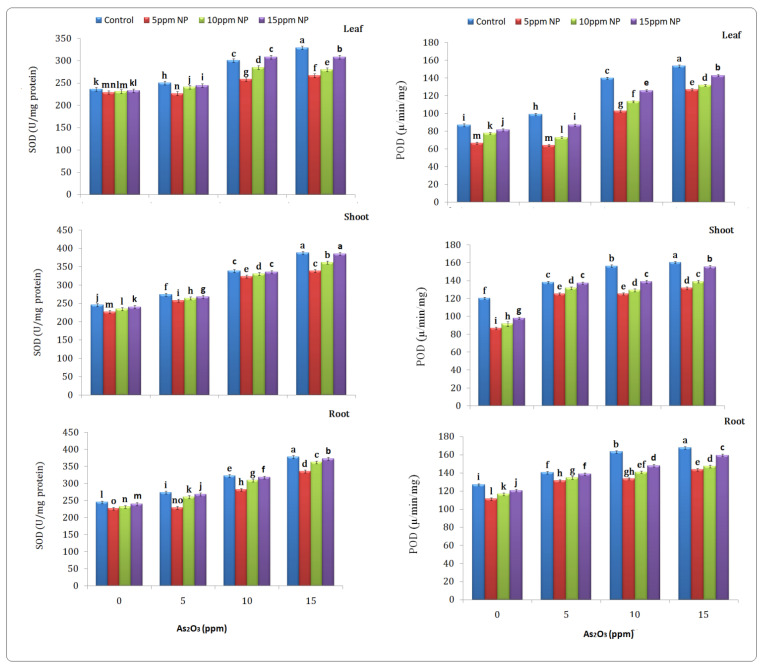
Effect of *Bacillus subtilis* synthesized iron oxide nanoparticles on SOD and POD content of rice (*Oryza sativa* L.) in arsenic-contaminated water. Different letters indicate significant differences (*p* < 0.05) between treatments.

**Figure 5:**
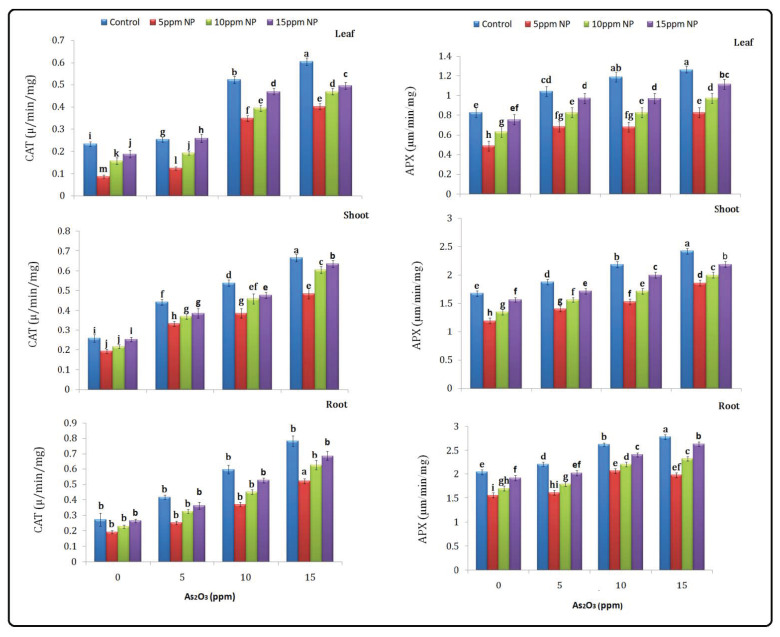
Effect of *Bacillus subtilis* synthesized iron oxide nanoparticles on CAT and APX content of rice (*Oryza sativa* L.) in arsenic contaminated water. Different letters indicate significant differences (*p* < 0.05) between treatments.

In plants under As treatment, different ROS are generated, which increased scavenging enzymes such as SOD, POD, CAT and APX. All enzymes showed different responses under different treatments such as arsenic, Fe_3_O_4_ NPs and (As + Fe_3_O_4_ NPs) combined treatment in plants (Figure 4) compared to control. Increased activity of the antioxidant enzyme was observed under arsenic stress. Anti oxidant enzymes SOD, POD, CAT and APX were higher at 15 ppm in the root, shoot and leaf under As stress (377.69, 388.07 and 329.16 µ/mg protein, (167.59, 160.47 and 153.47 µ/min/mg protein), (0.78, 0.66 and 0.60 µm/min/mg protein), (2.78, 2.42 and 1 µm/min/mg protein) as compared to the control. Furthermore, *Bacillus subtilis*-synthesized Fe_3_O_4_ NPs solution had no impact on enzyme activities at 5, 10 and 15 ppm. The As + Fe_3_O_4_ NPs combination at 5 ppm reduced the activities of these enzymes in the root, shoot and leaf (Figure 5).

### 3.5. Stree Bio-Markers

#### 3.5.1. Malondialdehyde (MDA)

**Figure 6:**
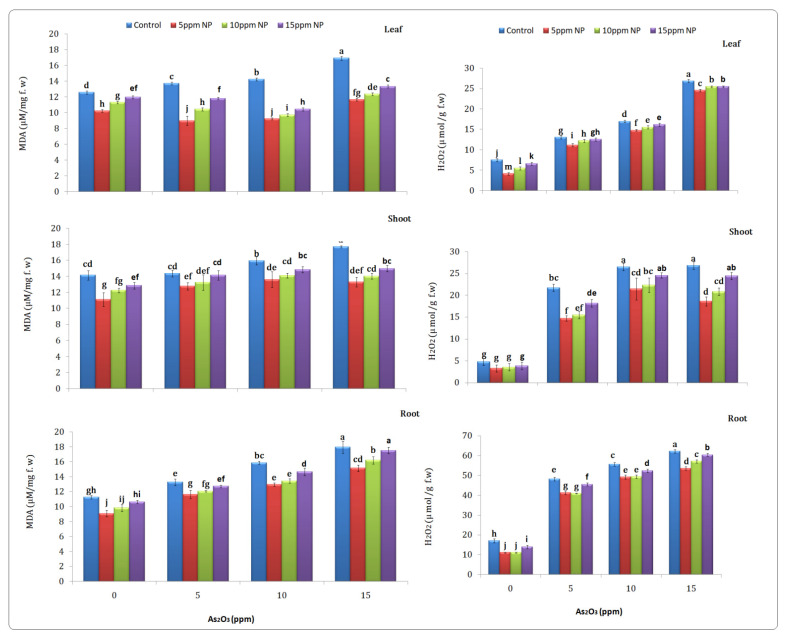
Effect of *Bacillus subtilis* synthesized iron oxide nanoparticles on MDA and H_2_O_2_ content of rice (*Oryza sativa* L.) in arsenic contaminated water. Different letters indicate significant differences (*p* < 0.05) between treatments.

Malondialdehyde is known as a biomarker of lipid per oxidation, and it determines the extent of oxidative damage to the membrane. MDA content increased in the root, shoot and leaf (17.95, 17.70 and 16.93 µM/mg) at 15 ppm As exposure. While As under *Bacillus subtilis*-synthesized Fe_3_O_4_ NPs reduced the MDA content in the root, shoot and leaf at 5 NPs + 15 As by (15.15, 14.14 and 11.67 µM/mg) as compared to arsenic treatment. However, arsenic and Fe_3_O_4_ NPs treatments alone showed a significant difference as compared to the control (Figure 6).

#### 3.5.2. Hydrogen Peroxide

Hydrogen peroxide is an evaluator of oxidative damage. The result showed that H_2_O_2_ content increased in the root, shoot and leaf at 15 ppm of NP solution (62.20, 26.78 and 26.86 µM/g f.wt) as compared to control (17.11, 7.79 and 7.53 µM/g f.wt). However, the 5 Fe_3_O_4_ NPs + 15 As combined treatment significantly decreased in the root, shoot, and leaf (53.57, 18.60 and 24.57 µM/g f.wt) as compared to the arsenic-alone treatment, respectively (Figure 6).

### 3.6. Determination of Arsenic Content in Plant Parts (Leaf, Shoot and Root)

**Figure 7:**
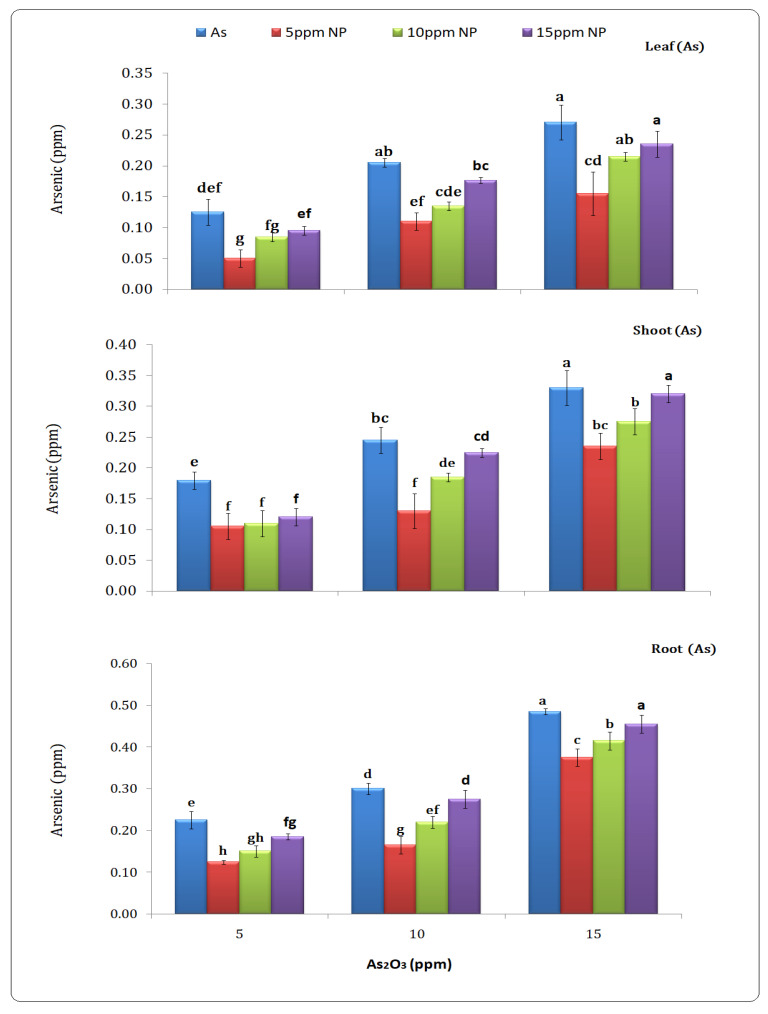
Effect of *Bacillus subtilis*-synthesized iron oxide nanoparticles on the arsenic content of rice (*Oryza sativa* L.) in arsenic contaminated water. Different letters indicate significant differences (*p* < 0.05) between treatments.

More arsenic accumulated in the roots, shoots and leaves of rice grown plants in arsenic-contaminated water. The arsenic level in the root (0.23, 0.30 and 0.48 ppm), shoot (0.18, 0.25 and 0.33 ppm), and leaf (0.13, 0.20 and 0.27 ppm) was determined at 5, 10 and 15 ppm. The results showed that iron oxide NPs remediate arsenic and reduce arsenic content in different parts of the plant at lower concentrations. Results observed the iron oxide NP treatment at 5 ppm decreased the arsenic content in the root (0.12, 0.16 and 0.37 ppm), the shoot (0.10, 0.13 and 0.23 ppm) and the leaf (0.05, 0.11 and 0.15 ppm), respectively (Figure 7).

## 4. Discussion

Currently, the methodology was designed to check the hypothesis that *Bacillus subtilis*-synthesized Fe_3_O_4_ nanoparticles could work as an adsorbent and alleviate the arsenic-induced stress in *Oryza Sativa* L. plants. Past examination has demonstrated that NPs have a minimal toxic impact on plants and are of importance in agriculture. When arsenic was not applied to the plant, the lower concentration of Fe NPs is not only less toxic but also enhances the growth of seedlings and is effective as a nano fertilizer. Higher concentrations of Fe_3_O_4_ NPs are toxic for plants because, through phosphatetransporters, it enters the plant and un couples the oxidative phosphorylation, reacts with thiol groups and inhibits the metabolic process [13].

It has been documented that arsenic disturbs the cell membrane permeability and function of nutrient transport, increasing them alone dialdehyde, the product of lipid peroxide as ion and electrolyte leakage in plants [35]. Results have revealed that cell injury levels were increased as the arsenic concentration increased; while the application of Fe_3_O_4_ NPs reduced the cell injury levels in plants (Figure 1) by enhancing the nutrient uptake and helping in the quenching of ROS in plants [49].

Photosynthetic pigments are them a in indicator of stress injury and also an important component of plant machinery. The results revealed that chlorophyll and carotenoid contents are inhibited under arsenic stress, while the application of iron oxide nanoparticles increased the content in the plant as compared to arsenics tress (Table 1). The reason behind this may be that under stressful conditions, the chlorophyll biosynthetic pathway is disrupted and enzyme activities are inhibited in the plant [50]. Similar results were also reported by [51] that photosynthetic pigments are enhanced by the addition of nanoparticles under stressful conditions because it enhances the production of chemical energy in photosynthetic systems and improves quantum yield in plants.

It has been documented that arsenic stress in rice decreases the total soluble protein content; on the other hand, the results revealed that *Bacillus subtilis*-synthesized Fe_3_O_4_ NPs increase the protein content under arsenic stress (Figure 2). In stressful conditions, the osmotic adjustment disturbed in plant and dehydration in different bio-molecule, and as a result, total soluble sugar content is reduced [52]. It has been documented that total soluble protein content is also reduced under arsenic stress due to modification in the specific site of amino acid, increase of photolytic enzyme and protein structured is turbance in plants [14] While Fe_3_O_4_ NPs increase the starch and osmotic adjustment, they also increase the nutrient accumulation and protect the protein from degradation [53]. The result revealed that (Figure 3), at the lower concentration, Fe_3_O_4_ NPs act as a micronutrient and increase the total soluble sugar and protein content in rice plants. Macromolecules are impaired in plants under heavy metal stress, these macromolecules enhance the protect ion mechanism in plants against reactive oxygen species. Certain metabolites and antioxidant enzymes play an important role in the survival of plants under stressful conditions. The Fe_3_O_4_ NPs did not increase the TSP directly but acted as nano fertilizer at lower concentrations and enhanced the germination process by increasing amylase activity and amino acid synthesis that in directly enhanced the total soluble sugar and total soluble protein content in plants.

Proline, an osmolyte, plays an important role in reducing the toxicity of metals [54]. These osmo-protectents, proline and glycine betain, protect the cytoplasm enzyme and organelles from damage and maintain the nutrient absorption in stressful conditions [55]. However, under arsenic treatment, the level was increased. It has been documented that the (Fe_3_O_4_ NPs + As) alleviated the harmful effect of metal by lowering the oxidative stress (Figure 3). Proline and Glycine Betain biosynthetic pathways are up-regulated by Fe_3_O_4_ NPs. These osmolytes are accumulated in plants and play a vital role in the alleviation of As stress. The result revealed that, similar to earlier reports, metal toxicity is alleviated in the presence of nanoparticles [56].

It has been documented that H_2_O_2_ and MDA levels were not increased by iron oxide nanoparticles. The results revealed that Fe_3_O_4_ NPs have the capacity to protect the plant from oxidative stress and inhibit the over production of ROS in plants (Figure 6). It has-been documented that iron ions in the form of Fe_3_O_4_ NPs can potentially act as micronutrients forrice plants. Additionally, under arsenic stress, the activity was higher and the plant needed greater protection to adapt with As-induced oxidative damage. Under arsenic stress, the redox homeostasis becomes imbalanced, the metabolic activities disturbed as a result of changes in per-oxidative activities in plants. In metal stress, similar results were also reported [57]. However, external application of some agents like nanoparticles could strengthen the defense system and provide a new sustainable approach to mitigate As stress [58].

Therefore, we suggest that particle size and growth medium are important for uptake. Fe_3_O_4_ NPs act as an adsorbent to decrease the arsenic toxicity. Probably, the main reason for restricting the entry of arsenic in to the plant is that the adsorption of As with Fe_3_O_4_ NPs and the increased size of particles in the solution that restricts them from entering the cell wall and move across the plasma membrane. We conclude that Fe_3_O_4_ nanoparticles enhance the plant’s growth and also help the plant survive with arsenic stress by limiting the uptake and mobility of arsenic. The beneficial action on arsenic when using 5 ppm Fe_3_O_4_ NPs in plant growth is that, at lower concentrations, the Fe_3_O_4_ NPs diffuse through nano-holes on the seed coat, it increases the water uptake, amylase activity and starch metabolism, which significantly improves the seed germination in green gram plants [52]. According to the findings of Mahakham [53], Fe_3_O_4_ NPs increase plant growth by regulating gibberellins and cytokinin, which are directly involved in cell division and elongation and in reducing ethylene production. The surface of iron oxide nanoparticles attracts the negatively-charged arsenic ion sand adsorbs them. Adsorption of arsenic on Fe_3_O_4_ NP surfaces depends on two steps. First, arsenic ions migrate from the bulk fluid phase to the outer nanoparticle surface for contact. Second, the electro static attraction between adsorbate (As) and adsorbent (Fe_3_O_4_ NPs) and this complex restricts the entry of arsenic into rice plants.

The results revealed that iron oxide NPs prevent arsenic from entering through plant parts (Figure 7). Lower concentrations of iron oxide nanoparticles NPs significantly reduced arsenic content, according to the observations. The surface chemistry and properties of nanoparticles were also revealed to restrict arsenic movement in plants [59]. Under arsenic stress, lipid per-oxidation activity increased, while iron oxide nanoparticles NPs reduced arsenic flow in water and plants and balanced ROS production, according to [60].

## 5. Conclusions

The results revealed that *Bacillus subtili* synthesized iron oxide nanoparticle increased the photosynthetic pigments and protein content and decreased the stress modulators in plants. In general, different stress parameters help the plant to adapt with mental stress. However, in this study decreased in different parameters due to the limited entry of arsenic into the plant, no detoxification machinery was required to protect the plant from stress. Nanoparticles uptake depends on the plant species, chemical composition, size, function, type, and stability of NPs. On the basic of the present investigation, it is being assumed that the association or aggregation of As + Fe_3_O_4_ NPs leads to reduced metal uptake in the plant.

## Figures and Tables

**Table 1:** Effect of *Bacillus subtilis*-synthesized iron oxide nanoparticles on photosynthetic pigment in the leaves and shoots of rice (*Oryza sativa* L.) in arsenic-contaminated water. Different letters indicate significant differences (*p* < 0.05) between treatments.

**Treatments (Leaf)**	**Chlorophyll (a)**		**Chlorophyll (b)**		**Total Pigments**		**Carotenoids**	
	**(mg/g)**		**(mg/g)**		**(mg/g)**		**(µg/g)**	
	**Mean**	**SD**	**Mean**	**SD**	**Mean**	**SD**	**Mean**	**SD**
Control	3.78	0.01 ^e^	2.44	0.03 ^e^	6.22	6.22 ^c^	1.54	0.00 ^e^
5 ppmNP	4.45	0.03 ^a^	2.85	0.04 ^bc^	7.3	7.33 ^a^	1.83	0.01 ^a^
10 ppmNP	4.35	0.35 ^b^	2.85	0.03 ^a^	7.2	7.23 ^a^	1.78	0.00 ^b^
15 ppmNP	4.25	0.04 ^c^	2.75	0.03 ^ab^	7	6.66 ^b^	1.69	0.01 ^c^
C + 5(As)	2.86	0.03 ^h^	2.34	0.04 ^e^	5.2	5.23 ^e^	1.23	0.01 ^j^
5 + 5(As)	3.85	0.03 ^d^	2.85	0.03 ^a^	6.7	6.73 ^b^	1.57	0.01 ^d^
5 + 10(As)	3.76	0.03 ^e^	2.65	0.04 ^cd^	6.41	6.41 ^c^	1.46	0.01 ^f^
5 + 15(As)	3.65	0.03 ^f^	2.55	0.03 ^d^	6.2	6.23 ^c^	1.38	0.01 ^g^
C + 10(As)	2.56	0.03 ^i^	1.55	0.03 ^h^	4.11	4.11 ^h^	1.1	0.01 ^m^
10 + 5(As)	3.63	0.03 ^f^	1.95	0.03 ^f^	5.58	5.57 ^d^	1.32	0.01 ^h^
10 + 10(As)	3.13	0.03 ^g^	1.85	0.04 ^g^	4.98	4.97 ^f^	1.28	0.00 ^i^
10 + 15(As)	2.85	0.03 ^h^	1.75	0.03 ^g^	4.6	4.63 ^g^	1.22	0.02 ^j^
C + 15(As)	2.05	0.03 ^l^	1.15	0.04 ^k^	3.2	3.23 ^j^	0.66	0.01 ^o^
15 + 5(As)	2.46	0.03 ^g^	1.34	0.04 ^i^	3.8	3.76 ^i^	1.18	0.01 ^k^
15 + 10(As)	2.25	0.04 ^k^	1.25	0.04 ^ij^	2.5	2.46 ^k^	1.12	0.00 ^l^
15 + 15(As)	2.06	0.01 ^l^	1.16	0.03 ^jk^	1.22	1.22 ^l^	0.98	0.01 ^n^
**Treatments (Shoot)**	**Chlorophyll (a)**		**Chlorophyll (b)**		**Total Pigments**		**Carotenoids**	
	**(mg/g)**		**(mg/g)**		**(mg/g)**		**(µg/g)**	
	**Mean**	**SD**	**Mean**	**SD**	**Mean**	**SD**	**Mean**	**SD**
Control	3.35	0.04 ^d^	1.55	0.04 ^g^	4.9	6.22 ^c^	1.34	0.04 ^c^
5 ppmNP	3.85	0.03 ^a^	2.42	0.01 ^a^	6.27	7.33 ^a^	1.47	0.01 ^a^
10 ppmNP	3.55	0.04 ^b^	2.39	0.00 ^ab^	6.15	7.23 ^a^	1.41	0.02 ^b^
15 ppmNP	3.45	0.03 ^c^	2.36	0.01 ^b^	5.81	6.66 ^b^	1.36	0.01 ^c^
C + 5(As)	3.25	0.04 ^e^	2.11	0.00 ^f^	5.36	5.23 ^e^	1.27	0.01 ^d^
5 + 5(As)	3.54	0.04 ^b^	2.28	0.01 ^c^	5.82	6.73 ^b^	1.45	0.02 ^a^
5 + 10(As)	3.45	0.04 ^c^	2.24	0.02 ^d^	5.85	6.41 ^c^	1.34	0.02 ^c^
5 + 15(As)	3.25	0.04 ^e^	2.17	0.06 ^e^	5.42	6.23 ^c^	1.3	0.05 ^c^
C + 10(As)	2.14	0.04 ^i^	1.2	0.01 ^jk^	3.6	4.11 ^h^	1.17	0.02 ^f^
10 + 5(As)	2.45	0.03 ^f^	1.36	0.02 ^h^	3.81	5.57 ^d^	1.34	0.02 ^c^
10 + 10(As)	2.35	0.04 ^g^	1.27	0.01 ^i^	3.62	4.97 ^f^	1.23	0.01 ^e^
10 + 15(As)	2.25	0.04 ^h^	1.24	0.04 ^ij^	3.49	4.63 ^g^	1.21	0.02 ^e^
C + 15(As)	1.74	0.04 ^k^	1.11	0.01 ^m^	2.85	3.23 ^j^	0.55	0.03 ^j^
15 + 5(As)	2.35	0.03 ^g^	1.24	0.02 ^ij^	3.59	3.76 ^i^	0.87	0.01 ^g^
15 + 10(As)	2.15	0.04 ^i^	1.16	0.01 ^kl^	3.31	2.46 ^k^	0.78	0.01 ^h^
15 + 15(As)	2.05	0.04 ^j^	1.15	0.04 ^lm^	3.2	1.22 ^l^	0.73	0.02 ^i^

## Data Availability

Not applicable.
